# Hypoxic Preconditional Engineering Small Extracellular Vesicles Promoted Intervertebral Disc Regeneration by Activating Mir‐7‐5p/NF‐Κb/Cxcl2 Axis

**DOI:** 10.1002/advs.202304722

**Published:** 2023-10-23

**Authors:** Hongxing Hu, Zhaojie Wang, Huiyi Yang, Yuxin Bai, Rongrong Zhu, Liming Cheng

**Affiliations:** ^1^ Key Laboratory of Spine and Spinal Cord Injury Repair and Regeneration Ministry of Education Department of Orthopedics Tongji Hospital Affiliated to Tongji University School of Medicine Tongji University Shanghai 200092 China; ^2^ Frontier Science Center for Stem Cell Research School of Life Science and Technology Tongji University Shanghai 200092 China; ^3^ Clinical Center for Brain and Spinal Cord Research Tongji University Shanghai 200092 China

**Keywords:** hypoxic preconditioning, intervertebral disc degradation, miRNA, small extracellular vesicles

## Abstract

Chronic low back pain (LBP) caused by intervertebral disc (IVD) degradation is a serious socioeconomic burden that can cause severe disabilities. Addressing the underlying pathogenic mechanisms of IVD degeneration may inspire novel therapeutic strategy for LBP. Herein, hypoxic preconditioning improves both the biological function of MSCs in hostile microenvironments and enhances the production of small extracellular vesicles (sEVs) with desirable therapeutic functions. In vitro results reveal that hypoxic preconditional engineering sEVs (HP‐sEVs) alleviate the inflammatory microenvironments of IVD degradation, enhance the proliferation of nucleus pulposus (NP) cells, and promote proteoglycan synthesis and collagen formation. Transcriptomic sequencing reveales the excellent therapeutic effects of HP‐sEVs in promoting extracellular matrix regeneration through the delivery of microRNA(miR)‐7‐5p, which further suppresses p65 production and thus the inhibition of Cxcl2 production. Moreover, in vivo results further confirm the robust therapeutic role of HP‐sEVs in promoting IVD regeneration through the same mechanism mediated by miR‐7‐5p delivery. In conclusion, this study provides a novel therapeutic strategy for treating IVD degradation and is thus valuable for understanding the mechanism‐of‐action of HP‐sEVs in IVD regeneration associated with chronic lower back pain.

## Graphical abstract

Hypoxic preconditional engineering small extracellular vesicles (HP‐sEVs) are injected into the intervertebral space. HP‐sEVs perform an anti‐inflammatory effect, promote the proliferation of nucleus pulposus (NP) cells, and promote proteoglycan synthesis, resulting in extracellular matrix regeneration. Successful intervertebral disc (IVD) regeneration is achieved via administering HP‐sEVs and this finding provides a promising method for IVD degradation treatment.

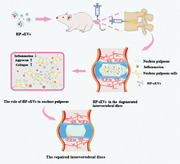

## 1 Introduction

Low back pain (LBP) is often caused by intervertebral disc (IVD) injury and degradation which can lead to disability and deteriorated quality of patients’ life.^[^ ^1^, ^2^ ^]^ IVD is a hydrated fibro‐cartilaginous tissue, composed of gel‐like nucleus pulposus (NP), annulus fibrosus (AF), and cartilage endplate and plays a crucial role in supporting spine stability and mobility. Specifically, proteoglycan‐rich gelatinous NP are rich in hydrated sulfated glycosaminoglycans and collagen type II, providing mechanical support and transmitting the external forces to AF.^[^ ^3^, ^4^ ^]^ Alterations in the biochemical and mechanical microenvironment of NP or AF are closely related to IVD degeneration as well as LBP.

Inflammatory responses triggered by mechanical loading, trauma, and aging are some of the most critical mechanisms that are responsible for the occurrence and progression of IVD degradation.^[^ ^5^ ^]^ The activation of inflammatory cytokines, including tumor necrosis factor‐alpha (TNF‐α), and interleukin‐1 (IL‐1), can result in a significant reduction of NP cell density and the homeostasis imbalance, which ultimately exacerbates extracellular matrix (ECM) degradation and hinder collagen synthesis.^[^ ^5^, ^6^ ^]^ Notably, ECM degradation may disturb the function of NP by inducing abnormal dehydration, compromised force dissipation, and impeded nutrient transportation, thereby accelerating the pathogenic process of IVD degradation.^[^ ^7^, ^8^ ^]^ Current treatments available for LBP have focused on symptomatic relief via nonsteroidal medication or invasive procedures, such as spinal stabilization and disc replacement.^[^ ^5^ ^]^ However, these therapeutic strategies do not fully address the underlying pathogenic mechanisms of IVD degeneration, limiting their efficacy in the restoration of the biological and mechanical functions of IVD.^[^ ^9^ ^]^ Therefore, new strategies to inhibit inflammatory microenvironment, and simultaneously regenerate proteoglycan and collagen type II are urgently required to treat IVD degradation.

Recent studies have demonstrated that local, regional injection of anti‐inflammatory small molecules, biologics, or stem cells can mitigate inflammation in IVD degeneration.^[^ ^10^ ^]^ Among various therapeutic approaches, administering mesenchymal stem cells (MSCs) is particularly promising due to its diverse secretome that targets heterogeneous inflammation pathways.^[^ ^11^, ^12^ ^]^ However, survival of injected MSCs remains a major challenge due to the harsh microenvironment (e.g., hypertonic, hypoxic, and avascular conditions) in IVD degradation.^[^ ^13^, ^14^ ^]^ Recent studies have reported that small extracellular vesicles (sEVs) derived from MSCs showed similar therapeutic power of MSCs in cartilage defects.^[^ ^15^ ^]^ Moreover, sEVs derived from MSCs possess anti‐inflammatory and trophic properties with satisfactory therapeutic potential for certain tissues, including bone, heart, skin, and other reproductive tissues.^[^ ^16^, ^17^, ^18^ ^]^

Notably, the IVD microenvironment represents low nutrient availability and special hypertonic and hypoxic conditions. Hypoxic preconditioning (HP), an adaptive response for sublethal hypoxia, is a promising strategy to enhance the survival of transplanted MSCs by lowering apoptosis after exposure to the inflammatory microenvironment.^[^ ^19^, ^20^ ^]^ HP enhances MSCs biological properties, such as proliferation, differentiation, and self‐renewal ability.^[^ ^21^, ^22^ ^]^ A previous study demonstrated that sEVs derived from the HP of dental pulp stem cells (DPSCs) could better suppress the macrophage inflammatory response and osteoclast genesis than native DPSCs. However, the effect of hypoxic preconditional engineering sEVs (HP‐sEVs) on IVD degradation remains unknown and the underlying mechanism needs further investigation.

**Scheme 1:**
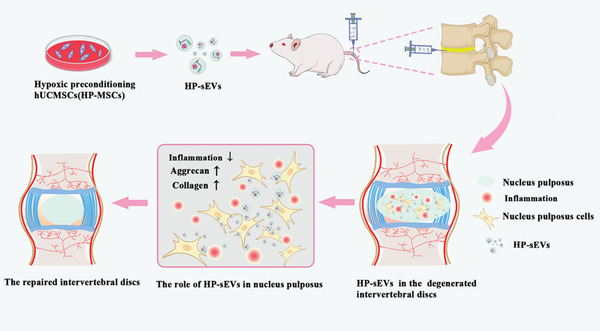
Graphical summary of the HP‐sEVs‐based IVD degradation theragnostic.

In this study, human umbilical cord MSCs (hUCMSCs) were cultured in a hypoxic atmosphere, and then through hypoxic conditioning, HP‐sEVs were extracted from the conditioned supernatant (**Scheme** **1**). After injection into the intervertebral space, HP‐sEVs were internalized by NP cells. HP‐sEVs performed an anti‐inflammatory effect, promoted the proliferation of NP cells, and promoted proteoglycan synthesis, and collagen formation, resulting in ECM regeneration. Successful IVD regeneration was achieved via administering HP‐sEVs and this finding provides a promising method for IVD degradation treatment.

## 2 Results

### 2.1 Identification of hUCMSCs and HP‐sEVs

**Figure 1:**
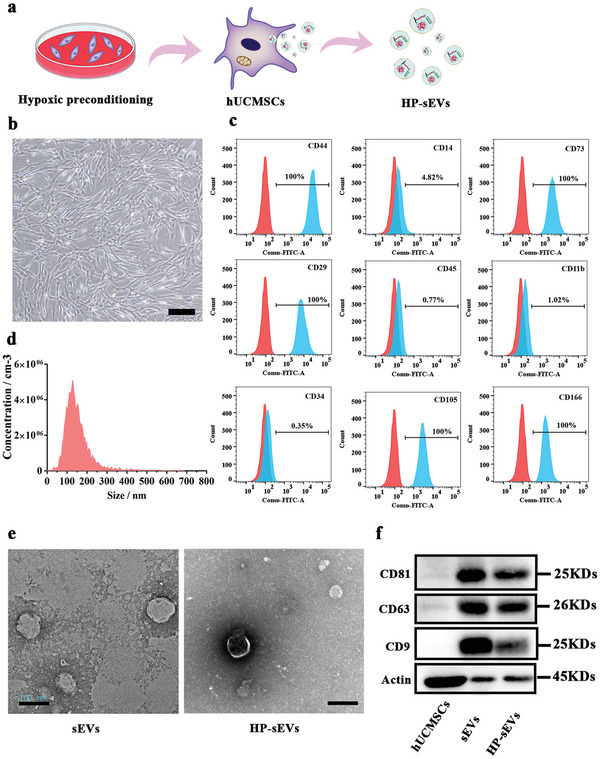
Identification of hUCMSCs and HP‐sEVs. a) Scheme of HP‐sEVs isolation. b)The spindle‐like morphology of hUCMSCs. c) Surface markers of hUCMSCs were analyzed via flow cytometric analysis. Red curves represent the control group and blue curves denote the surface of hUCMSCs. d) Particle size of HP‐sEVs characterized by nanoparticle tracking analysis (NTA). e) TEM was used to visualize the morphology of sEVs and HP‐sEVs. Scale bar: 100 nm. f) Surface markers of sEVs and HP‐sEVs were characterized using Western Blot.

hUCMSCs were cultured under hypoxic conditions for 6 h. HP‐sEVs were isolated from the conditioned medium of hypoxic preconditioned hUCMSCs (**Figure** **1**a). Microscopic images revealed the typical spindle‐like morphology of hUCMSCs (Figure 1b). Moreover, hUCMSCs showed multiple differentiation functions including chondrogenic, adipogenic, and osteogenic differentiation potential (Figure S1, Supporting Information). Surface markers of hUCMSCs were evaluated using flow cytometric analysis (Figure 1c). The results showed that the positive markers, including CD44, CD166, CD29, CD73, and CD105 were expressed in 100% of the cells. The negative markers CD14, CD45, CD34, and CD11b were expressed on 4.82%, 0.77%, 0.35% and 1.02% of cells, respectively. Subsequently, we cultured hUCMSCs in the hypoxic atmosphere and collected the conditioned supernatants. To estimate whether a hypoxic atmosphere was successfully created by the AnaeroPack MicroAero system, western blotting was used to test the hypoxia‐inducible factor‐1 alpha (HIF‐1α) protein. HIF‐1α protein expression was increased when hUCMSCs were cultured in the hypoxic condition for 6 h (Figure S2, Supporting Information). The average diameter of HP‐sEVs was 130.4 nm and the concentration was 2.1 × 10^11^ particles per mL(Figure 1d). HP‐sEVs were isolated from the conditioned supernatants and then characterized by TEM (Figure 1e). HP‐sEVs and sEVs exhibited typical cup‐like shapes with double‐layer membrane structures. HP‐sEVs and sEVs have similar diameter sizes of about 100 nm. Western blot analysis showed that both sEVs and HP‐sEVs were positive for the exosomal surface marker including CD9, CD63, and CD81. In contrast, hUCMSCs lacked these surface markers (Figure 1f). These results demonstrate the successful extraction of HP‐sEVs from the conditioned medium.

### 2.2 HP‐sEVs Suppress the Inflammatory Microenvironment Induced by TNF‐α

**Figure 2:**
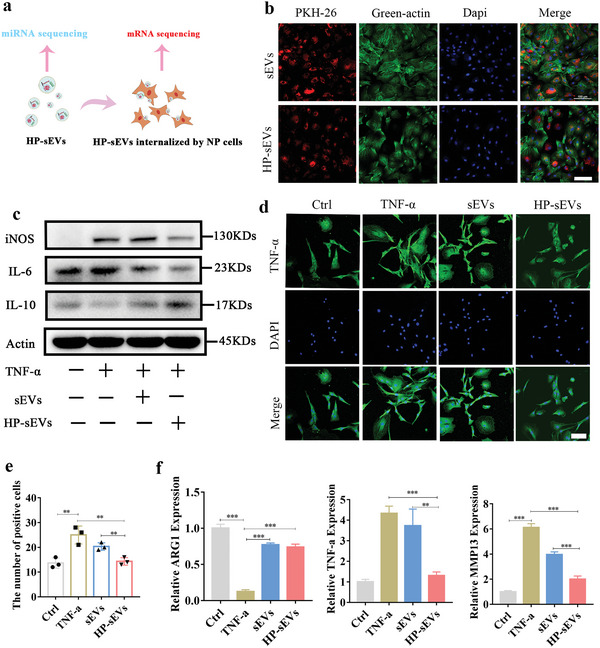
Anti‐inflammatory effect of HP‐sEVs. a) Mode pattern of co‐incubation of nucleus pulposus cells (NP cells) and HP‐sEVs. b) Fluorescence image of NP cells uptaken PKH‐26 labeled HP‐sEVs. Green actin was used to stain the cytoskeleton. Scale bar: 100 µm. c) Inflammatory proteins including iNOS, IL‐6, IL‐10 were estimated by western blotting. d) Immunofluorescent of TNF‐α in NP cells. In the sEVs and HP‐sEVs group, NP cells were treated with sEVs or HP‐sEVs (20 µg mL^−1^), respectively, following TNF‐α (50 ng mL^−1^) stimulation for 24 h. Scale bar: 100 µm. e) Quantitative analysis of TNF‐α positive cells. Immunofluorescence‐positive cells were analyzed by Image J software (*n* = 3, ^*^ *p* < 0.05, ^**^ *p* < 0.01). f) qPCR analysis of inflammatory‐related genes, including ARG1, TNF‐α, and MMP13 (*n* = 3, ^***^ *p* < 0.001).

To validate the role of HP‐sEVs in NP cells in vitro, NP cells were co‐incubated with PKH‐26 labeled HP‐sEVs for 24 h (**Figure** **2**a). As illustrated in Figure 2b, the cytoskeleton of NP cells was stained in green fluorescence by actin. HP‐sEVs were stained in red fluorescence using PKH‐26, which was absorbed and distributed around the nuclei. Inducible nitric oxide synthase (iNOS) and interleukin‐6 (IL‐6) are key mediators of immune activation and inflammation.^[^ ^23^, ^24^ ^]^ Interleukin‐10 (IL‐10) is an anti‐inflammatory cytokine.^[^ ^25^ ^]^ Macrophages and monocytes could generate TNF‐α during acute inflammation. As an important inflammatory cytokine, TNF‐α is accountable for various signaling events within cells, resulting in necrosis.^[^ ^26^, ^27^ ^]^ In this study, TNF‐α was used to stimulate NP cells to mimic the inflammatory environment in vitro. Our results showed that HP‐sEVs suppressed iNOS and IL‐6 protein expression following TNF‐α stimulation, while upregulated the expression of IL‐10 protein induced by TNF‐α (Figure 2c). Immunofluorescent was performed to observe TNF‐α protein expression in NP cells (Figure 2d). The images from confocal laser microscopy revealed that TNF‐α stimulation significantly upregulated the expression of TNF‐α protein in NP cells, while HP‐sEVs inhibited TNF‐α expression in NP cells. Consistently, inflammatory genes, including matrix metallopeptidase 13 (MMP13) and TNF‐α in the HP‐sEVs group, were significantly down‐regulated compared to the levels in the TNF‐α group, while HP‐sEVs significantly increased the expression level of arginase 1(ARG1). Inhibition of MMP13 is an effective strategy to decelerate both ECM loss and osteoarthritis progression.^[^ ^28^ ^]^ Taken together, HP‐sEVs inhibit the inflammatory environment induced by TNF‐α and possess anti‐inflammatory roles in vitro.

### 2.3 HP‐sEVs Promote the Proliferation of NP Cells and Facilitate Proteoglycan Synthesis and Collagen Formation

**Figure 3:**
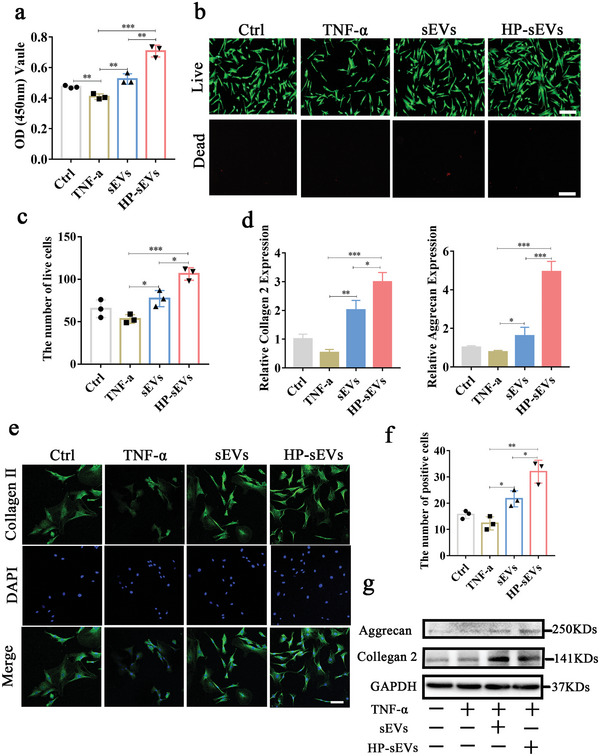
HP‐sEVs promote the proliferation of NP cells and facilitate proteoglycan synthesis and collagen formation. a) CCK8 analysis of NP cells treated with sEVs and HP‐sEVs under inflammatory environments (stimulated with 50 ng mL^−1^ TNF‐α). b) Fluorescence images of NP cells stained with calcein AM (live cells, green fluorescence) and PI (dead cells, red fluorescence). In the sEVs and HP‐sEVs groups, NP cells were treated with sEVs or HP‐sEVs (20 µg mL^−1^), respectively, following stimulation with 50 ng/mL TNF‐α for 24 h. Scale bar: 250 µm. c) Quantitative analysis of live cells (*n* = 3, ^*^ *p* < 0.05, ^***^ *p* < 0.001). d) RNA expression levels of collagen type II, and aggrecan were determined using qPCR (*n* = 3, ^*^ *p* < 0.05, ^***^ *p* < 0.001). e) Immunofluorescent of collagen type II in NP cells stimulated with sEVs or HP‐sEVs under inflammatory environments. In the sEVs and HP‐sEVs groups, NP cells were treated with TNF‐α (50 ng mL^−1^) and sEVs or HP‐sEVs (20 µg mL^−1^) for 24 h. Scale bar: 100 µm. f) Quantitative analysis of collagen type II positive NP cells. Immunofluorescence‐positive cells were analyzed by Image J software (*n* = 3, ^*^ *p* < 0.05, ^***^ *p* < 0.001). g) Aggrecan and collagen type II proteins were evaluated using Western Blot.

To investigate whether HP‐sEVs affect the viability of NP cells in an inflammatory microenvironment in vitro, NP cells were cultured with HP‐sEVs following TNF‐α stimulation. The results showed that sEVs and HP‐sEVs significantly promoted the proliferation of NP cells; HP‐sEVs show better effect on enhancing the viability of NP cells (**Figure** **3**a). Subsequently, a live and dead staining kit was used to estimate the survival condition of NP cells under the inflammatory environment. HP‐sEVs treatment significantly increased the number of live cells, indicating that HP‐sEVs enhanced the proliferation potential of NP cells (Figure 3b,c). NP is mainly constituted by proteoglycans and collagen type II.^[^ ^29^ ^]^ Aggrecan is a member of the proteoglycan family of extracellular matrix proteins and loss of aggrecan is a known characteristic of IVD degradation. qPCR was carried out to further evaluate the role of HP‐sEVs on IVD regeneration (Figure 3d). The results revealed that sEVs and HP‐sEVs upregulated the mRNA expression levels of aggrecan and collagen type II compared to those in the control group. Furthermore, immunofluorescent images from confocal laser microscopy revealed that the production of collagen type II was significantly enhanced in the NP cells treated with HP‐sEVs plus TNF‐α (Figure 3e,f). Consistent with the immunofluorescent result, the protein expression level of collagen type II and aggrecan was significantly increased in the HP‐sEVs group compared to those in the TNF‐α group (Figure 3g).

### 2.4 HP‐sEVs Are Conducive to Regenerating NP Tissues and Repairing the Degraded IVD

**Figure 4:**
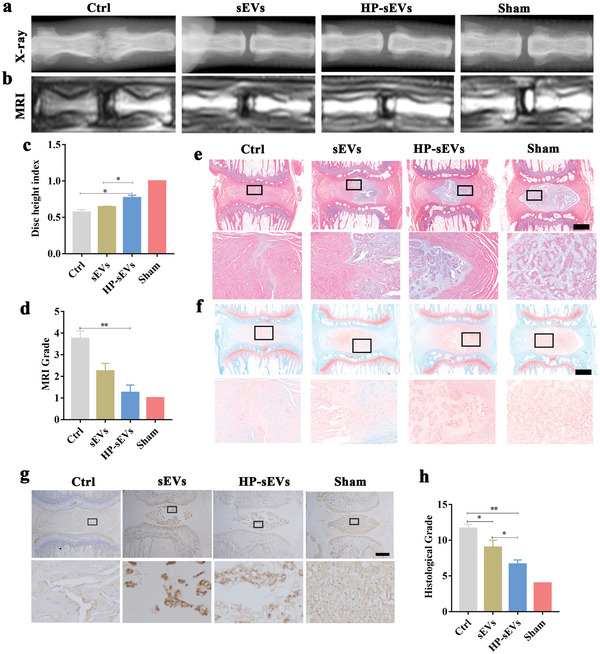
Imageological and histological evaluation of IVD repaired by HP‐sEVs. a) X‐ray and b) MRI analyses of rat coccygeal vertebrae. c) Disc height index (DHI) of different groups (*n* = 3, ^*^ *p* < 0.05). d) MRI grade at 6 weeks after surgery (*n* = 3, ^**^ *p* < 0.01). e) HE staining of IVD. Scale bar:250 µm. f) Safranin O/fast green staining of IVD. Scale bar: 250 µm. g) Histological grade of different treatments at 6 weeks after surgery(*n* = 3, ^*^ *p* < 0.05, ^**^ *p* < 0.01). h) Immunohistochemical staining of collagen type II. Scale bar: 250 µm.

We established an SD rat disc puncture model to investigate the potential of HP‐sEVs for IVD degradation treatment in vivo. sEVs and HP‐sEVs were injected into the IVD after puncturing with a 21G needle. PBS was used as a control. X‐ray imaging of the control group revealed that IVD space was obviously narrow, and osteophytes and bone spurs were generated from the vertebral body. IVD space in the sEVs group showed a little decrease compared to that in the sham group. In contrast, the HP‐sEVs group showed a similar IVD space to the sham group, indicating that HP‐sEVs have a better therapeutic effect on IVD degradation than sEVs in vivo (**Figure** **4**a,c). Notably, IVD space was closely associated with ECM content, which was mainly composed of proteoglycans and collagen type II. The high signal intensity of T2‐weighted MRI is commonly used to evaluate proteoglycans in the IVD. Proteoglycans are lost, and water retention is impaired during IVD degradation, resulting in dehydration of NP tissue. Herein, a low signal of NP and the black disc was observed in the control group, while T2‐weighted images in the HP‐sEVs group showed a white high signal, similar to that in the sham group (Figure 4b,d). Hematoxylin and Eosin (HE) and safranin O/fast green staining were performed to evaluate the regeneration of newly formed NP tissues (Figure 4e–g). Few regenerated NP tissues were observed in the control group. In contrast, the HP‐sEVs group showed more regenerated NP tissues than the sEVs group. Moreover, Giemsa‐positive tissues in the HP‐sEVs group were decreased compared to those in the sEVs group, indicating that HP‐sEVs could inhibit the proliferation of monocytes in vivo (Figure S3, Supporting Information). Immunohistochemical staining showed that the HP‐sEVs group had more type II collagen than the sEVs group, which was similar to the sham group (Figure 4h). In brief, administering HP‐sEVs revealed superior biological functions in promoting NP tissue regeneration and repairing degraded IVD in vivo.

### 2.5 Discovery of HP‐sEVs‐Associated miRNAs by Small RNA Sequencing

**Figure 5:**
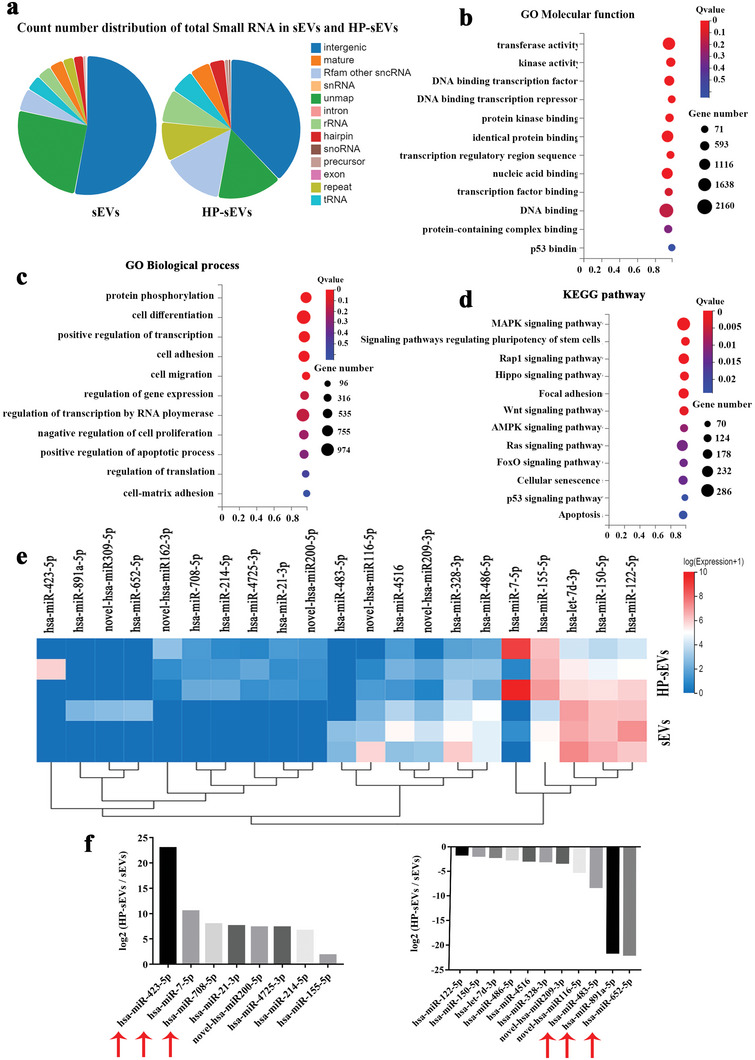
Discovery of HP‐sEVs‐associated miRNAs by small RNA sequencing. a) Count number distribution of sEVs and HP‐sEVs. b) Gene Ontology (GO) analysis for molecular function of all differentially expressed miRNAs. c) GO analysis for the biological process of differentially enriched miRNAs. d) Enriched pathways for target genes of miRNAs enriched within HP‐sEVs in KEGG pathways. e) Heatmap of clustered dysregulated miRNA expression profiles with microarray in HP‐sEVs compared to that in sEVs control. f) MiRNA expression levels in HP‐sEVs compared to that in sEVs control.

MicroRNA(miRNA) is instrumental in regulating gene expression by binding the 3′‐UTR or amino acid coding sequences of the target gene.^[^ ^30^ ^]^ To further verify the underlying mechanism of anti‐inflammatory, proteoglycans synthesis and collagen formation mediated by HP‐sEVs, miRNA expression profiles of sEVs and HP‐sEVs were measured by high‐throughput small RNA sequencing. The count number distribution of the total small RNA of sEVs and HP‐sEVs was different (**Figure** **5**a). Gene ontology (GO) analysis of molecular function and biological processes for differentially expressed miRNAs was performed to thoroughly investigate the effect of miRNA involved in the IVD regeneration (*p* < 0.05, log_2_ fold change >1). The potential target genes of the differentially expressed miRNAs in HP‐sEVs were associated with biological processes including protein phosphorylation, cell differentiation, regulation of transcription, and cell adhesion (Figure 5b,c). The results from the Kyoto Encyclopedia of Genes and Genomes (KEGG) pathway enrichment analysis (*p* < 0.05, log_2_ fold change >1) were based on the potential target genes involved in a broad range of signaling pathways, such as MAPK signaling pathway, the signaling pathway regulating pluripotency of stem cells and RAP1 signaling pathway (Figure 5d). Furthermore, we selected the first few differentially expressed miRNAs (high‐ and low‐expression miRNAs). Figure 5e,f shows a clustered heatmap and the normalized expression levels of the first few differentially expressed miRNAs (high‐ and low‐expression miRNAs).

### 2.6 Transcriptome Profiling of Optimized HP‐sEVs‐Regulated NP Cells Function and Mechanism

**Figure 6:**
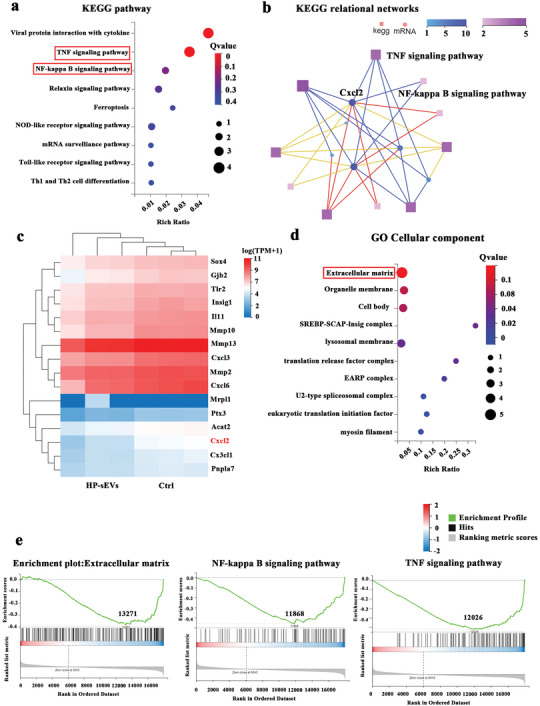
Transcriptome profiling of optimized HP‐sEVs regulates NP cells' function and mechanism. a) KEGG pathway enrichment analysis and b) KEGG relation networks based on HP‐sEVs regulated significant target genes. c) Heatmap distribution of HP‐sEVs regulating significant target genes. In the HP‐sEVs group, NP cells were treated with HP‐sEVs (20 µg mL^−1^) following TNF‐α (50 ng mL^−1^) stimulation for 24 h. In the control (Ctrl) group, NP cells were treated with TNF‐α (50 ng mL^−1^). d) GO analysis for the cellular component of differential target genes. e) Gene set enrichment analysis (GSEA) analysis of ECM, NF‐κB signaling pathway, and TNF‐α signaling pathway.

To explore the potential mechanism of HP‐sEVs‐regulated NP cell function, mRNA profiles of HP‐sEVs‐stimulated NP cells were examined and comparatively analyzed by mRNA sequencing. KEGG pathway enrichment analysis (*p* < 0.05, log_2_ fold change >1) revealed that differentially expressed genes (DEGs) were mainly associated with TNF‐α/NF‐κB/Cxcl2 signaling pathway (**Figure** **6**a). A previous study reported that methamphetamine triggers a neuroinflammatory response by upregulating TNF‐α and then activating NF‐κB in human brain endothelial cells.^[^ ^31^ ^]^ KEGG relation networks and heatmap distribution revealed that Cxcl2 was one of the DEGs and showed closely associated with the TNF‐α/NF‐κB signaling pathway, indicating Cxcl2 may be the key downstream gene of the TNF‐α/NF‐κB signaling pathway (Figure 6b,c). Cxcl2 was a chemokine associated with proinflammatory function and its downregulation inhibited the recruitment of polymorphonuclear cells, which were responsible for the elimination of monocytogenes during infection.^[^ ^32^ ^]^ Gene ontology (GO) analysis of cellular components based on DEGs showed that ECM was different between the HP‐sEVs and control groups, indicating that HP‐sEVs may repair IVD by promoting ECM regeneration (Figure 6d). TNF‐α/NF‐κB signaling pathway and ECM were altered in gene set enrichment analysis (GSEA), suggesting that HP‐sEVs may contribute to ECM regeneration by regulating the TNF‐α/NF‐κB/Cxcl2 signaling pathway (Figure 6e).

### 2.7 HP‐sEVs Activated TNF‐α/NF‐κB/Cxcl2 Signaling Pathway by Delivering miR‐7‐5p

**Figure 7:**
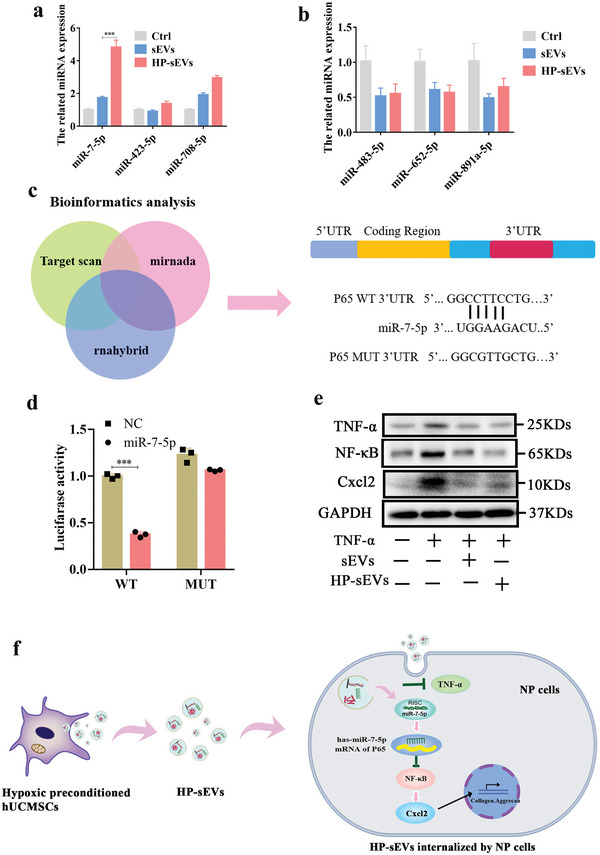
HP‐sEVs activate TNF‐α/NF‐κB/Cxcl2 signaling pathway by delivering miR‐7‐5p. a) The first three differentially high expressed miRNAs expression levels in NP cells (*n* = 3, ^***^ *p*＜0.001). b) The first three differentially low expressed miRNAs expression levels in NP cells treated with sEVs or HP‐sEVs . c) Bioinformatic analysis was used to predict the potential target sequences of miR‐7‐5p. d) HEK293T cells were transfected with luciferase reporter plasmids WT or MUT 3′‐UTR of P65 and miR‐7‐5p, and luciferase activity was detected by the Dual‐Luciferase Reporter Assay System (*n* = 3, ^***^ *p* < 0.001). e) TNF‐α/NF‐κB/Cxcl2 signaling pathway‐related proteins in NP cells were measured by western blot. f) Schematic diagram of the molecular mechanism of HP‐sEVs affection for NP cells.

To investigate the miRNA HP‐sEVs activating downstream signaling pathways, NP cells were cultured with sEVs or HP‐sEVs, and then qPCR was performed to measure the expression level of HP‐sEVs enriched miRNAs in NP cells (the first three differentially high or low expressed miRNAs, marked with red arrow in Figure 5e,f). The expression of miR‐7‐5p was significantly upregulated than other miRNAs, indicating that HP‐sEVs activate downstream signaling pathways by delivering miR‐7‐5p (**Figure** **7**a,b). Transcriptome profiling revealed that HP‐sEVs may contribute to ECM regeneration by regulating the NF‐κB/Cxcl2 signaling pathway. To further validate whether HP‐sEVs regulated the NF‐κB/Cxcl2 signaling pathway by transferring miR‐7‐5p, the potential target gene of miR‐7‐5p was predicted via bioinformatic analysis. Among the predicted target genes, p65 is one of five components of the NF‐κB transcription factor family and plays an important role in the transactivation of several target genes involved in immunity, inflammation, and proliferation.^[^ ^33^ ^]^ The database showed that miR‐7‐5p could bind the 3′‐UTR region of p65 (Figure 7c). To experimentally investigate whether miR‐7‐5p directly binds p65, luciferase reporter plasmids, including WT‐psicheck2‐p65 and MUT‐psicheck2‐p65 were constructed and transfected into 293T cells. The luciferase value was reduced following miR‐7‐5p stimulation, while the reaction was terminated through mutating 3′‐UTR regions of p65 (Figure 7d). The above results demonstrate that miR‐7‐5p targets the 3′‐UTR region of the p65 gene and suppresses the expression of p65.

Furthermore, we estimated the related proteins of the NF‐κB/Cxcl2 signaling pathway, such as TNF‐α, NF‐κB, phosphorylated NF‐κB (p‐NF‐κB), and Cxcl2. Western blot results showed that TNF‐α stimulation upregulated the levels of TNF‐α, p‐NF‐κB, and Cxcl2, while protein expression was decreased in the presence of HP‐sEVs (Figure 7e). Hypoxic preconditioned hUCMSCs secrete numerous HP‐sEVs. HP‐sEVs were internalized by NP cells, upregulating the expression level of miR‐7‐5p in NP cells and suppressing the level of TNF‐α. The increased miR‐7‐5p directly targeted 3′‐UTR of p65 via RNA‐induced silencing complex and inhibited the level of p65. The inhibitory p65 signal inhibited Cxcl2 production, resulting in aggrecan formation, collagen II deposition, and inflammatory downregulation (Figure 7f).

### 2.8 miR‐7‐5p Silencing Alleviates HP‐sEVs‐Mediated Anti‐Inflammatory Effect, Proteoglycans Synthesis, and Collagen Formation

**Figure 8:**
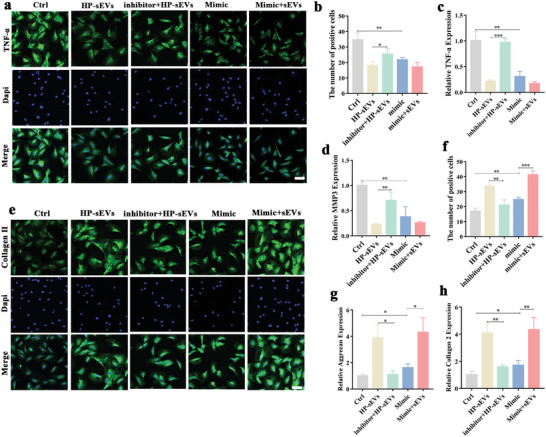
miR‐7‐5p silencing alleviates HP‐sEVs‐mediated anti‐inflammatory effect, proteoglycans synthesis, and collagen formation. a) Immunofluorescence of TNF‐α in NP cells under inflammatory environments. In the control group, NP cells were stimulated with TNF‐α for 24 h. In the inhibitor + HP‐sEVs group, NP cells were transfected with miR‐7‐5p inhibitor and treated with HP‐sEVs. In the mimic group, NP cells were transfected with miR‐7‐5p. Scale bar: 100 µm. b) Quantitative analysis of TNF‐α positive NP cells (*n* = 3, ^*^ *p* < 0.05, ^**^ *p* < 0.01). c,d) qPCR analysis of inflammatory‐related genes including TNF‐α and MMP3 (*n* = 3, ^**^ *p* < 0.01, ^***^ *p* < 0.001). e) Immunofluorescence of collagen type II in NP cells under inflammatory environments. Scale bar: 100 µm. f) Quantitative analysis of collagen type II positive NP cells. Immunofluorescence‐positive cells were analyzed by Image J software (*n* = 3, ^**^ *p* < 0.01, ^***^ *p* < 0.001). g,h) qPCR analysis of proteoglycans synthesis‐related genes, including aggrecan and collagen type II (*n* = 3, ^*^ *p* < 0.05, ^**^ *p* < 0.01).

We investigated whether miR‐7‐5p was essential and involved in HP‐sEVs‐mediated IVD regeneration in vitro. Either a miR‐7‐5p inhibitor or miR‐7‐5p mimic was transfected into NP cells to deplete or enhance, respectively, the expression of mature miRNAs. As shown in **Figure** **8**a,b, miR‐7‐5p inhibitor treatment significantly diminished the HP‐sEV‐mediated downregulation of TNF‐α protein in NP cells. Synthetic miR‐7‐5p administration (Group mimic) significantly reduced TNF‐α expression compared to that in the control group. Consistently, miR‐7‐5p inhibitor administration significantly attenuated the downregulation of inflammatory‐related genes including MMP3 and TNF‐α (Figure 8c,d). Moreover, HP‐sEVs‐mediated collagen type II formation and proteoglycans synthesis were reversed by miR‐7‐5p inhibitor administration, whereas synthetic miR‐7‐5p significantly promoted the expression of aggrecan and collagen type II (Figure 8e–h), indicating that miR‐7‐5p treatment showed a similar effect as HP‐sEVs in promoting collagen type II formation and proteoglycans synthesis. Additionally, NF‐κB activator administration significantly diminished the miR‐7‐5p‐mediated downregulation of TNF‐α and IL‐6 genes in NP cells (Figure S4, Supporting Information). Taken together, HP‐sEVs possess anti‐inflammatory effects and contribute to NP regeneration, mainly via delivering miR‐7‐5p.

### 2.9 Administered Agomir‐7‐5p Contributes to the Repair of IVD Degradation

**Figure 9:**
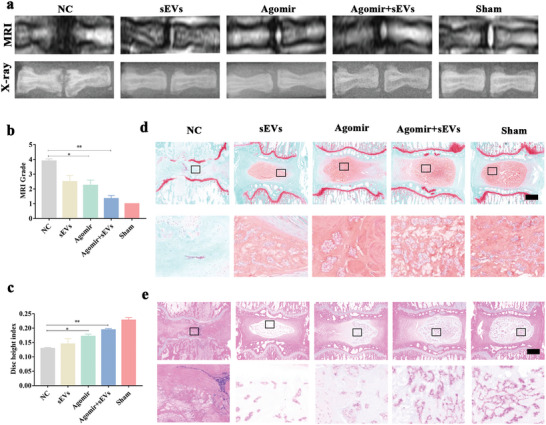
Administered agomir‐7‐5p contributes to the repair of degraded IVD. a) X‐ray and MRI analyses of rat coccygeal vertebrae. NC denotes the invalid sequence of agomir‐7‐5p. b) MRI grade at 6 weeks after surgery (*n* = 3, ^*^ *p* < 0.05, ^**^ *p* < 0.01). c) Disc height index (DHI) of different groups (*n* = 3, ^*^ *p* < 0.05, ^**^ *p* < 0.01). d) Safranin O/fast green staining of IVD. Scale bar: 250 µm. e) HE staining of IVD. Scale bar: 250 µm.

To demonstrate that miR‐7‐5p mediates the effect of HP‐sEVs in promoting NP regeneration and repair of degraded IVD in vivo, we used agomir‐7‐5p in an SD rat disc puncture model. Agomir‐7‐5p with or without sEVs was administered into intervertebral space. MRI revealed a high white signal in the agomir group, while the NC group showed a low signal of NP and black discs. Moreover, X‐ray imaging of the control group revealed that IVD space was narrow, and osteophytes and bone spurs were generated from the vertebral body. Agomir‐7‐5p injection significantly increased the IVD space, reducing friction between the vertebrae (**Figure** **9**a–c). Consistently, Safranin O/fast green and HE staining showed that few of the regenerated NP tissues were observed in the NC group, while agomir‐7‐5p group showed more regenerated NP tissues than in the NC and sEVs group, indicating that agomir‐7‐5p contributed to repaired IVD degradation (Figure 9d,e). In summary, the collective results demonstrate that HP‐sEVs promote NP regeneration and repair degraded IVD in vivo, mainly via transferring miR‐7‐5p.

## 3 Discussion

IVD structural integrity is vital for spine stability and mobility.^[^ ^8^ ^]^ Therefore, IVD degradation may compress the nerve roots, resulting in strained mobility of spinal joints and inducing chronic lower back pain.^[^ ^34^ ^]^ Current therapies for IVD degradation include conservative treatments and surgical methods.^[^ ^5^ ^]^ Conservative treatments temporarily alleviate the pain via physical therapy and nonsteroidal anti‐inflammatory drugs, while surgical techniques such as total disc replacement are often invasive.^[^ ^5^, ^35^ ^]^ However, it has been a challenging task in terms of simultaneous regeneration of NP tissue and restoration of the biological and mechanical function of IVD. Herein, we adapted a hypoxic preconditioning strategy to harvest hUCMSCs‐derived sEVs (HP‐sEVs) for enhanced treatment of IVD degeneration and mitigation of LBP. In vitro and in vivo assays supported the robust role of HP‐sEVs for the inhibition of the inflammatory microenvironment after IVD degeneration, which further regenerated the ECM microenvironment such as proteoglycan and collagen type II by transferring miR‐7‐5p and inhibiting the NF‐κB/Cxcl2 axis. The superior effects of HP‐sEV over conventional EVs are further proven in a rat IVD degeneration model with the improvement of NP tissue regeneration and thus the IVD regeneration.

Administering MSCs is considered a promising therapy for IVD degradation due to its favorable anti‐inflammatory properties.^[^ ^36^ ^]^ However, the hostile microenvironment in IVD degradation contains special hypertonic, hypoxic, and avascular conditions, limiting the broad clinical applications of MSC therapy.^[^ ^13^, ^14^ ^]^ Notably, sEVs realized similar therapeutic efficacy of MSC‐based therapies in terms of the regeneration of cartilaginous tissues.^[^ ^15^ ^]^ Previous studies have suggested that sEVs derived from MSCs pre‐conditioned by LPS may have better regulatory abilities for macrophage polarization and thus more effective suppression of inflammation, as compared to sEVs derived from non‐LPS‐treated MSCs.^[^ ^37^ ^]^ In this study, we aimed to establish a novel functional sEV‐based strategy based on hypoxic preconditioning for the effective treatment of IVD degradation. HP is a powerful, endogenous protective mechanism that improves the biological functions of MSCs in harsh microenvironments.^[^ ^19^, ^20^, ^21^, ^22^ ^]^ Nevertheless, the therapeutic effect and the underlying mechanism of action of HP‐sEVs remain to be investigated in the context of IVD degeneration.

The key to achieving IVD regeneration is to design an effective therapeutic therapy that targets the underlying pathogenic mechanisms of IVD degradation. The critical issues in the progression of IVD degradation have included inflammatory responses, progressive cell apoptosis, and deterioration in anabolic activities and cellular functions.^[^ ^38^ ^]^ To address these challenges, we compared the morphological and functional differences between sEVs and HP‐sEVs. Characterization of sEVs using NTA and TEM suggested that there lacks significant morphological differences between the sEVs and HP‐ sEVs in terms of their size, shape, or electron density. Moreover, we have also shown that HP‐sEVs demonstrate improved anti‐inflammatory function as compared to conventional sEVs. Additionally, HP‐sEVs also showed superior outcomes in terms of promoting NP cell proliferation, proteoglycan formation, and collagen type II synthesis. Proteoglycans are key biomacromolecules regulating cell proliferation, differentiation, and ECM turnover.^[^ ^39^ ^]^ Collagen is an important component of the ECM and provides structural and mechanical support to disc cells.^[^ ^7^ ^]^ The collective findings in this study strongly support the view that HP‐sEVs could improve the degenerated IVD microenvironment by inhibiting the inflammatory response and decreasing catabolic activities. Meanwhile, HP‐sEVs can increase anabolic activity by promoting ECM regeneration and facilitating NP cell proliferation, resulting in a favorable IVD microenvironment and accelerated regeneration.

MiRNAs are non‐coding RNAs and also key mediators of the therapeutic function of sEVs.^[^ ^40^, ^41^ ^]^ Recent studies have reported that sEVs promoted vascularized bone regeneration by transferring miR‐23a‐3p.^[^ ^30^ ^]^ However, a differential analysis of the miRNA profile between HP‐sEVs and sEVs and the miRNA‐mediated effects of promoting NP regeneration remains to be investigated. In this study, RNA sequencing indicated the differential miRNA profile of HP‐sEVs. Specifically, miR‐423‐5p, miR‐7‐5p, and miR‐708‐5p were significantly enriched in HP‐sEVs compared to that in sEVs, whereas miR‐483‐5p, miR‐891a‐5p, and miR‐652‐5p levels were lower in HP‐sEVs than in sEVs. We further demonstrated that miR‐7‐5p expression levels were significantly upregulated compared to those of other miRNAs in NP cells treated with HP‐sEVs. Moreover, HP‐sEVs‐mediated collagen type II formation and proteoglycans synthesis were reversed by miR‐7‐5p inhibitor administration. In contrast, synthetic miR‐7‐5p showed a biological function similar to that of HP‐sEVs in promoting the expression of aggrecan and collagen type II. These findings indicated the strong anti‐inflammatory effects of HP‐sEVs that are crucial for NP regeneration which is likely achieved by the delivery of miR‐7‐5p existent in HP‐sEVs. Subsequently, our transcriptome profiling results revealed the activation of the NF‐κB/Cxcl2 signaling pathway in the HP‐sEVs‐treated NP cells, suggesting that HP‐sEVs may contribute to ECM by regulating NF‐κB/Cxcl2 signaling pathway. Moreover, we demonstrated that miR‐7‐5p can bind to the 3′‐UTR region of p65, which is one of five components of the NF‐κB transcription factor family. In addition, miR‐7‐5p mimetics inhibited the production of p65, whereas the mutant miR‐7‐5p increased p65 production. NF‐κB signaling pathway regulates inflammatory and oxidative responses.^[^ ^42^, ^43^ ^]^ Herein, we verified that HP‐sEVs transferred miR‐7‐5p into NP cells. Upregulated miR‐7‐5p targets the p65 gene and suppresses p65 production. The inhibitory p65 signal further inhibits Cxcl2 production, resulting in inflammatory response downregulation, aggrecan formation, and collagen II deposition.

In summary, our study demonstrates the superior functions of HP‐sEVs in promoting IVD regeneration compared to that of normal sEVs. In addition, we highlighted the underlying mechanism by which HP‐sEVs suppressed inflammatory response and promoted IVD regeneration. Hypoxic preconditioning upregulated the production of miR‐7‐5p in sEVs, and the enriched level of exosomal miR‐7‐5p significantly promoted the therapeutic effect of HP‐sEVs on IVD degradation. HP‐sEVs alleviated the inflammatory microenvironment involved in IVD degradation, promoted NP cell proliferation, and enhanced proteoglycan synthesis and collagen formation, thus promoting IVD regeneration by activating the miR‐7‐5p/NF‐κB/Cxcl2 axis. Therefore, we envisage that HP‐sEV‐based therapy may serve as a promising treatment for IVD degradation as well as lower back pain. Moving forward, a more accurate analysis of the spatiotemporal distribution of HP‐sEVs after injection is required to understand its pharmacokinetic profiles. Rigorous optimization of dosimetry of HP‐sEVs for fully mitigating LBP in both small and large animal models, and large‐scale HP‐sEVs production would also be essential before its clinical translation.

## 4 Experimental Section

### Cell Isolation and Culture

Nucleus pulposus cells (NP cells) were isolated from NP tissues of 8 weeks old Sprague–Dawley rats. According to the previous protocol,^[^ ^44^ ^]^ the rats were euthanized by injecting excessive pentobarbital sodium. Subsequently, NP tissues were obtained and transferred to a sterile PBS. NP tissues were minced to a size of 1 mm^3^ roughly and then digested by 0.1% collagenase type II (Invitrogen, 17 101 015, USA) which was dissolved in DMEM/F12(Corning, 10 092 001, USA). After digested for 4 h, the solution was filtrated by a 70 µm cell strainer. The cells were obtained after removing the collagenase solution at 316 RCF. NP cells were cultured in DMEM/F12 containing 10% fetal bovine serum (FBS, Gibco, 10099–141, USA). The above procedures were completed in a sterile environment.

### hUCMSCs Characterization

hUCMSCs were cultured in basal medium (DAKAWE, 6 114 021, China) supplemented serum analog EliteGro‐Adv (Elitecell Biomedical, EPA‐050, Canada). hUCMSCs at a density of 10 × 10^4^ cells were stimulated via the primary antibodies (Cyagen, HUXMX‐09011, China) for 30 min in a dark environment. The cells were centrifuged for 5 min at 1000 rpm, followed by washing with PBS. The centrifuge tubes were obtained from NEST Biotechnology. Finally, flow cytometry was used to analyze the cells after co‐incubating with FITC IgG antibody for 10 min.

The multipotency of hUCMSCs was evaluated by inducing MSCs into chondrogenic, adipogenic, and osteogenic cells. hUCMSCs were incubated in chondrogenic, adipogenic, and osteogenic differentiated medium (Cyagen, China).

### Isolation and Identification of HP‐sEVs

A hypoxic cultural environment was established by the AnaeroPack method according to the previous methods.^[^ ^45^, ^46^ ^]^ In brief, AnaeroPack (Cat# D07, Mitsubishi Gas Chemical Company, Japan) was placed into a sealed air‐tight container to mimic the hypoxic atmosphere environment. Then, hUCMSCs were cultured in a 10 cm dish and placed into a sealed air‐tight container. HP‐sEVs were isolated from the conditional supernatants of hUCMSCs cultured in the hypoxic atmosphere. hUCMSCs were cultured in a medium containing exosomes‐free FBS (System Biosciences, USA, 50A‐1) at 37 °C, regular (21% O_2_) or hypoxic (1% O_2_) condition. As previously described,^[^ ^47^ ^]^ sEVs were obtained from a conditional medium by gradient centrifugation. Briefly, the supernatants were centrifuged at 300 g for 10 min, 2000 g for 10 min, and 10 000 g for 30 min at 4 °C to clear away cell debris. Subsequently, the conditional medium was re‐centrifuged at 1 00 000 g for twice at 4 °C and then the sediment was resuspended with PBS to obtain sEVs or HP‐sEVs. A transmission electron microscope (TEM, G2 spirit BioTWIN by Tecnai) was used to observe the morphology of sEVs and nanoparticle tracking was used to measure the particle concentration and distribution.

### PKH‐26 Staining Assay

According to the manufacturer's instructions, HP‐sEVs and sEVs were labeled through PKH‐26 (Sigma, Germany). Briefly, 4 µL PKH‐26 dye was added into 500 µL Dilution C solution to obtain the mixture solution. Subsequently, HP‐sEVs were incubated with the above mixture solution in a dark environment for 30 min. The process of staining was stopped by 1% bovine serum albumin. Then, the mixture containing sEVs was centrifuged at 1 00 000 g at 4 °C. The labeled HP‐sEVs were resuspended by sterile PBS. NP cells were cultured with PKH‐26 labeled HP‐sEVs for 24 h and then fixed by 4% paraformaldehyde (PFA). Subsequently, NP cells were incubated with DAPI. The photograph was observed via microscopy.

### Western Blot Analysis

Radio‐immunoprecipitation assay buffer (RIPA, Amizona Scientific LLC, China) was used to lysis and extract protein. The BCA method was used to measure the protein concentration. After separation by SDS‐PAGE gel, proteins were transferred to polyvinylidene difluoride (PVDF) membranes. Subsequently, PVDF membranes were blocked via 5% bovine serum albumin (BSA) and then co‐incubated with primary antibodies overnight at 4 °C. Then, PVDF membranes were washed with tris‐buffered saline with tween‐20 (TBST, UElandy, China) three times. PVDF membranes were incubated with secondary antibodies for 1 h at room temperature. The protein bands were observed with ChemiDoc XRS+ Gel Imaging System (Bio‐Rad, USA). Primary antibodies included anti‐NF‐κB (CST, 8242, USA), anti‐p‐NF‐κB (CST, 3033, USA), Cxcl2(Abcam, ab275879, USA), IL‐6(CST, 12 912, USA), IL‐10(Abcam, ab133575, USA), collagen II (Abcam, ab34712, USA), Aggrecan (Abcam, ab3778, USA) and β‐actin (Forevertech Biotechnologies, 20270, China).

### Quantitative RT‐PCR Analysis

Trizol reagent was used to extract the total RNA of the cells. RNA was extracted by SPARKeasy Cellular RNA Extraction Kit (Sparkjade Biotechnology, China). Then, cDNA was obtained after reverse transcription of RNA. According to the manufacturer's protocols, cDNA was amplified with TB Green Premix EX Taq II (Takara, RR820A, Japan). The primers used in qPCR are shown in Table S1 (Supporting Information). Reverse transcription of miRNA into cDNA was performed using miDETECT A Track miRNA qRT‐PCR Starter Kit (RiboBio, China). Internal controls for mRNA and miRNA were GAPDH and U6 RNA, respectively. The results were analyzed by the 2^−ΔΔCT^ quantitative method. Data were shown as mean ± SD of biological triplicates.

### Cell Proliferation Assay

The proliferation ability of NP cells was evaluated by cell counting kit‐8(CCK‐8, KeyGEN, China) and LIVE/DEAD Fixable dead cell stains (Invitrogen, USA). Briefly, NP cells cultured with a density of 5000 cells were cultured in DMEM/F12 medium containing TNF‐α (50 ng mL^−1^), sEVs, or HP‐sEVs (20 µg mL^−1^) in a 96‐well plate for 1 day. Subsequently, CCK8 was added in the medium and co‐cultured for 2–4 h. Then, the viability of NP cells was measured via absorbance values at 450 nm.

NP cells with a density of 2 × 10^4^ were implanted in a 24‐well plate. sEVs or HP‐sEVs (20 µg mL^−1^) were co‐incubated with the cells for 24 h. Calcein‐AM and PI were diluted in DMEM/F12 at a ratio of 1:2000. NP cells were incubated with the mixture medium for 30 min. Subsequently, calcein‐AM and PI solution were used to detect live cells (green fluorescence) and dead cells (red fluorescence), respectively.

### Immunofluorescence Staining

NP cells were incubated with sEVs or HP‐sEVs for 3 days. Then, the cells were fixed using 4% PFA and then washed with PBS. Subsequently, NP cells were treated with 0.25% Triton X‐100 (Elabscience Biotechnology, China) and then blocked with 5% donkey serum. NP cells were treated with primary antibodies including TNF‐α (Abcam, ab1793, USA) and collagen II (Abcam, ab34712, USA) overnight at 4 °C. After being washed by PBS, NP cells were conjugated with a fluorescent secondary antibody (Invitrogen, USA) and stained with DAPI for 5 min at room temperature. Finally, NP cells were visualized via the confocal microscope (Carl Zeiss, Germany). Immunofluorescence‐positive cells were analyzed by Image J software.

### miRNA Inhibitor Transient Infection

MiRNA inhibitors and mimics were purchased from RiboBio company. According to the manufacturer's instructions, when NP cells were cultured at 60−70% density, miRNA inhibitors or mimics were transfected into NP cells via lipofectamine 3000 (Invitrogen, L3000008). After being transfected for 1 day, the transfection medium was removed. Then, the growth medium contained tumor necrosis factor‐alpha (TNF‐α) and HP‐sEVs were added.

### Animal Experiment

An ethical review committee of Tongji Hospital affiliated with Tongji University reviewed and approved the details of animal experiments (ID:2022‐DW‐(003)). According to the previous protocol,^[^ ^29^ ^]^ Sprague–Dawley rats were anesthetized and then disinfected with iodine. IVD between Co5/6 was exposed by slitting the skin. Then, a needle (21 g) was used to puncture the center of the discs, rotated for 5 s then the position was maintained for 30 s. Ten microliters sEVs or HP‐sEVs were injected into the disc. Four groups were fabricated in this study. 1) control (10 µL PBS was injected into the disc), 2) sEVs group, 3) HP‐sEVs group, and 4) Sham group. The rats in the sham group were only incised in the outer layer of skin at the corresponding site without needle puncture. Animals were divided into four groups in this study to further demonstrate that miR‐7‐5p mediated the effect of HP‐sEVs in vivo. 1) NC (injected invalid sequence of agomir‐7‐5p (100 nm)), 2) sEVs group, 3) agomir group (injected agomir‐7‐5p), 4) agomir+sEVs group (injected agomir‐7‐5p (100 nm) and sEVs), and 5) Sham group.

### X‐Ray and MRI Examination

The therapeutic effect of sEVs and HP‐sEVs for IVD degradation was evaluated by X‐ray and MRI at 6 weeks post‐surgery. In brief, the rats were anesthetized, and then their tail was exposed in a straight line on a molybdenum target radiation‐imaging device. The exposure parameters of the X‐ray included 32 kV and 10 s duration. X‐ray images were measured by Image J software to obtain disc height index (DHI) values. DHI values were calculated by the degraded disc divided by the normal disc and represented the disc height change of the degraded disc compared with the normal disc. MRI was carried out by Vero 1.5T system (Siemens, Germany). T2‐weighted images were captured in the coronal plane. The parameter of MRI was that visual field, 80 × 80 mm; slice thickness:1.4 mm; repetition time, 4000 ms; echo time, 96 ms. T2‐weighted images were graded into I–IV according to the modified Thomson classification method.^[^ ^48^ ^]^

### Histology and Immunohistochemical Analysis

Animal specimens were placed in 10% formalin for 2 days. Subsequently, the samples were decalcified and then embedded in paraffin. Six micrometers thick sections of tissue samples were obtained by paraffin slicing machine. Then, the tissue sections were stained with Safranin‐O & fast green, hematoxylin and eosin (H&E). Immunohistochemical staining was according to the previous protocol.^[^ ^49^ ^]^ In brief, the sections of samples were repaired with sodium citrate solution to achieve epitope retrieval. Subsequently, the samples were incubated with primary antibodies including collagen II (Abcam, USA) overnight at 4 °C. On the next day, the sections were co‐incubated with a second antibody and then stained with hematoxylin.

### Statistical Analysis

All results are expressed as mean ± standard deviation. The unpaired Student's *t*‐test was used to evaluate the difference between the two groups. One‐way analysis of variance (ANOVA) was used to evaluate the difference between multiple groups. Differences were considered statistically significant when *p* < 0.05. Data were expressed as the mean ± standard deviation of three replicates. The *t*‐test was used to compare the mean beta values of each group.

## Conflict of Interest

The authors declare no conflict of interest.

## Author Contributions

H.H. and Z.W. contributed equally to this work. H.H. performed investigation, data curation, methodology, and wrote‐original draft. Z.W. performed data curation, investigation, and methodology. H.Y. performed investigation and data curation. Y.B. performed investigation. R.Z. performed conceptualization, supervision, and project administration. L.C. performed conceptualization, supervision, and project administration.

## Supporting information

Supporting Information

## Data Availability

The data that support the findings of this study are available from the corresponding author upon reasonable request.
