# Supplementary material for: Hypoxic Preconditional Engineering Small Extracellular Vesicles Promoted Intervertebral Disc Regeneration by Activating Mir‐7‐5p/NF‐Κb/Cxcl2 Axis
Source: Adv Sci (Weinh). 2023 Oct 23;10(35):2304722. doi: 10.1002/advs.202304722 (PMC10724439; doi:10.1002/advs.202304722)
Supplement: Supplementary file 1 — Supporting Information [file ADVS-10-2304722-s001.pdf]

ADVANCED SCIENCE
Open Access

Supporting Information

for *Adv. Sci.*, DOI 10.1002/advs.202304722

Hypoxic Preconditional Engineering Small Extracellular Vesicles Promoted Intervertebral Disc Regeneration by Activating Mir-7-5p/NF-Kb/Cxcl2 Axis

Hongxing Hu, Zhaojie Wang, Huiyi Yang, Yuxin Bai, Rongrong Zhu and Liming Cheng**

Hypoxic preconditional engineering small extracellular vesicles promoted intervertebral disc regeneration by activating miR-7-5p/NF-κB/Cxcl2 axis

Hongxing Hu[a 1], Zhaojie Wang[a,b 1], Huiyi Yang[a], Yuxin Bai[a], Rongrong Zhu[a,b*], Liming Cheng[a,c*]

a. *Key Laboratory of Spine and Spinal Cord Injury Repair and Regeneration, Ministry of Education, Department of Orthopedics, Tongji Hospital Affiliated to Tongji University, School of Medicine, Tongji University, Shanghai 200092, China.*

b. *Frontier Science Center for Stem Cell Research, School of Life Science and Technology, Tongji University, Shanghai 200092, China.*

c. *Clinical Center for Brain and Spinal Cord Research, Tongji University, Shanghai 200092, China.*

1. The authors contributed equally to this work.

**Corresponding authors:*

Rongrong Zhu: Email: rrzhu@tongji.edu.cn.

Liming Cheng: Email: limingcheng@tongji.edu.cn.

Supplementary Figures

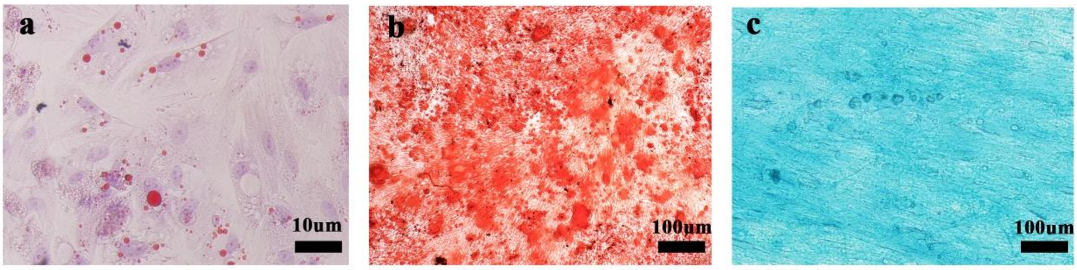

Supplementary Figure 1. hUC-MSCs show muti-lineage differentiation of adipogenesis (a), osteogenesis (b) and chondrogenesis (c).

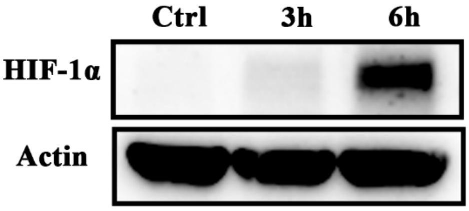

Supplementary Figure 2: Western blotting is implemented to evaluate the expression levels of HIF-1α in hUC-MSCs.

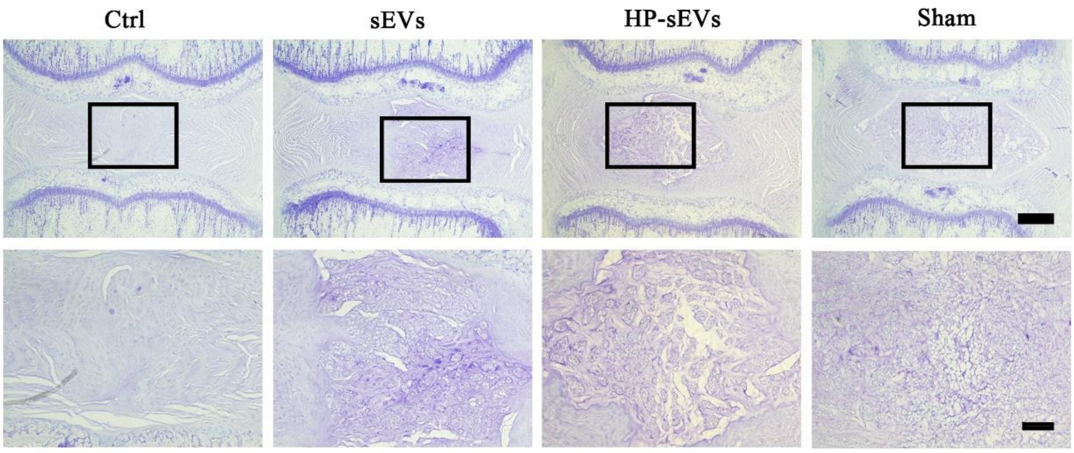

Supplementary Figure 3: Giemsa staining of IVD. Scale bar: 100 μm.

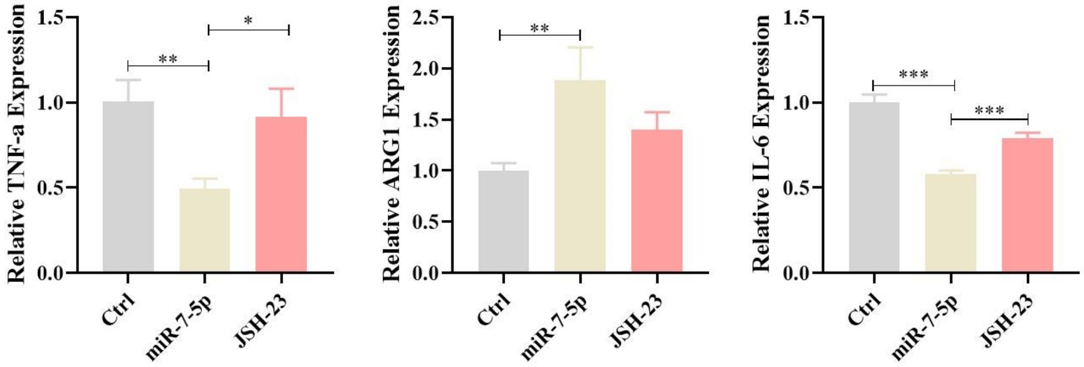

Supplementary Figure 4: qPCR analysis of inflammatory-related genes, including ARG1, TNF-α, and IL-6. The ctrl group means NP cells were stimulated with TNF-α (50 ng/mL) for 24 hours. In the miR-7-5p group, NP cells were transfected with miR-7-5p, following TNF-α stimulation for 24 hours. In the NF-κB activator group, NP cells were transfected with miR-7-5p and treated with NF-κB activator, following TNF-α stimulation for 24 hours (n=3, *P<0.05, **P<0.01, ***P<0.001).

Supplementary Table 1

Gene	Sequences
MMP-13	F-5'-GGCCAGAACTTCCCAACCA-3'
	R-5'-ACCCTCCATAATGTCATACCC-3'
TNF-α	F-5'-CAGGCGGTGCCTATGTCTC-3'

	R-5'- CGATCACCCCGAAGTTCAGTAG-3'
Arg1	F-5'- CTCCAAGCCAAAGTCCTTAGAG-3'
	R-5'- TGTAGACCATGTAGTTGAGGTCA-3'
MMP-3	F-5'-GGCCTGGAACAGTCTTGGC-3'
	R-5'- TGTCCATCGTTCATCATCGTCA-3'
GAPDH	F-5'-AGGTCGGTGTGAACGGATTTG-3'
	F-5'-TGTAGACCATGTAGTTGAGGTCA-3'
